# Hepatic Hydatid Cyst with Biliary Communication

**DOI:** 10.4269/ajtmh.25-0283

**Published:** 2025-09-04

**Authors:** Venkatesh Vaithiyam, Sanjeev Sachdeva, Siddharth Srivastava

**Affiliations:** Department of Gastroenterology, GB Pant Hospital and Associated Maulana Azad Medical College, New Delhi, India

**Figure 1.**
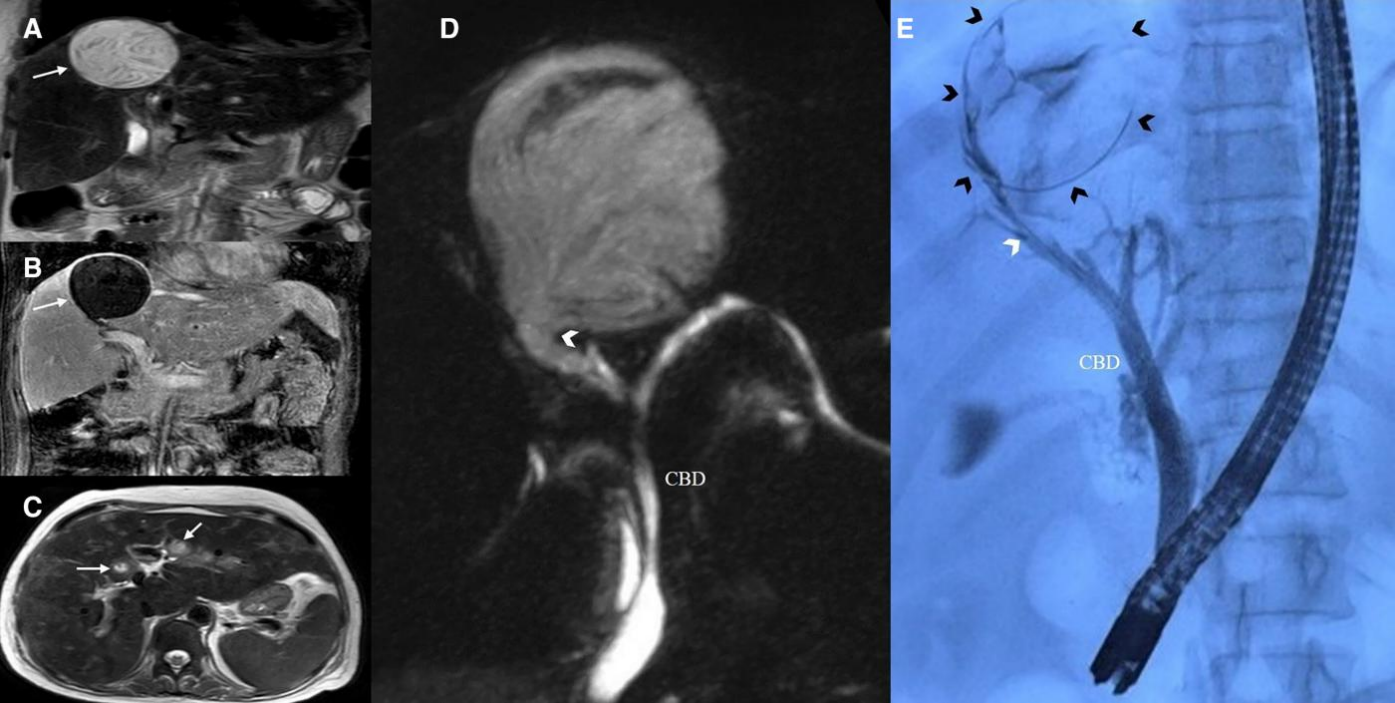
**(A–C)** MRI showed a well-defined, rounded, hyperintense lesion with thin internal septations in segment VIII, suggestive of a hepatic hydatid cyst. **(D)** Magnetic resonance cholangiopancreatography revealed communication between the segment VIII lesion and right hepatic duct. **(E)** Cholangiography revealed extravasation of contrast medium into the cyst, confirming cystobiliary communication.

A 45-year-old woman residing in a rural part of northern India as an agricultural laborer, having low socio-economic status with no comorbidities, presented with right upper-quadrant pain for six months. No fever, vomiting, jaundice, or awareness of an abdominal lump was presented. Physical examination showed pallor, and the rest of the systemic examination was unremarkable. Laboratory findings showed iron-deficiency anemia, with a total leukocyte count of 21,000/mm^3^ with elevated absolute eosinophil count of 1,980/mm,^3^ and elevated alkaline phosphatase (506 IU, normal value [40 –20 IU]) and gamma-glutamyl transferase levels (225 IU, normal value [<85 IU]). Hydatid serology on ELISA was strongly positive. Magnetic resonance imaging showed a well-defined, rounded, hyperintense lesion with thin internal septations in segment VIII, suggestive of a hepatic hydatid cyst (Figure 1). Magnetic resonance cholangiopancreatography revealed communication between the segment VIII lesion and right hepatic duct (Figure 1). Surgical excision was planned and the patient underwent endoscopic retrograde cholangiopancreatography to rule out cystobiliary communication. Cholangiography revealed extravasation of contrast medium into the cyst, confirming cystobiliary communication (Figure 1). Endoscopic papillotomy and balloon trawling were performed; no membranes were retrieved, and a 7Fr X 12 cm plastic biliary stent was placed. The patient is planned for definitive surgical removal of the cyst.

Hydatid liver disease comprises 50–70% of echinococcosis cases, with intrabiliary communication occurring in up to 17% of hepatic hydatid infections.^1^^,^^2^ Cystobiliary communication may be occult or frank, with signs ranging from asymptomatic biliary dilation to cholangitis and obstructive jaundice. Early recognition guided by clinical suspicion and radiological findings, with timely intervention, can prevent severe complications.^3^
